# Allele Compensation in Tip60^+/−^ Mice Rescues White Adipose Tissue Function *In Vivo*

**DOI:** 10.1371/journal.pone.0098343

**Published:** 2014-05-28

**Authors:** Yuan Gao, Nicole Hamers, Maryam Rakhshandehroo, Ruud Berger, John Lough, Eric Kalkhoven

**Affiliations:** 1 Molecular Cancer Research, Center for Molecular Medicine, University Medical Centre Utrecht, Utrecht, The Netherlands; 2 Netherlands Metabolomics Center, Leiden, The Netherlands; 3 Department of Cell Biology, Neurobiology and Anatomy and the Cardiovascular Center, Medical College of Wisconsin, Milwaukee, Wisconsin, United States of America; Northern Institute for Cancer Research, United Kingdom

## Abstract

Adipose tissue is a key regulator of energy homestasis. The amount of adipose tissue is largely determined by adipocyte differentiation (adipogenesis), a process that is regulated by the concerted actions of multiple transcription factors and cofactors. Based on *in vitro* studies in murine 3T3-L1 preadipocytes and human primary preadipocytes, the transcriptional cofactor and acetyltransferase Tip60 was recently identified as an essential adipogenic factor. We therefore investigated the role of Tip60 on adipocyte differentiation and function, and possible consequences on energy homeostasis, *in vivo*. Because homozygous inactivation results in early embryonic lethality, Tip60+/− mice were used. Heterozygous inactivation of Tip60 had no effect on body weight, despite slightly higher food intake by Tip60+/− mice. No major effects of heterozygous inactivation of Tip60 were observed on adipose tissue and liver, and Tip60+/− displayed normal glucose tolerance, both on a low fat and a high fat diet. While Tip60 mRNA was reduced to 50% in adipose tissue, the protein levels were unaltered, suggesting compensation by the intact allele. These findings indicate that the *in vivo* role of Tip60 in adipocyte differentiation and function cannot be properly addressed in Tip60+/− mice, but requires the generation of adipose tissue-specific knock out animals or specific knock-in mice.

## Introduction

The relationship between obesity and its complications, such as type 2 diabetes and cardiovascular diseases, has firmly established adipose tissue as a key regulator of glucose and lipid metabolism [1]. Adipose tissue regulates metabolism through at least two different mechanisms: the storage and release of lipids, and the secretion of so-called adipokines, which function in an endocrine or paracrine fashion. Expansion of adipose tissue, as seen in obese individuals, not only affects the storage of lipids as triglycerides in lipid droplets, but also results in qualitative and quantitative changes in a number of adipokines [2]. The amount of mature adipocytes is largely determined by the differentiation of fibroblast-like mesenchymal stem cells into adipocytes, a process called adipogenesis [1], [3]. Adipogenesis is regulated by a cascade of transcription factors, ultimately leading to the induction of the transcription factor peroxisome proliferator activator receptor γ (PPARγ) [1], [4]. In general, adipogenic transcription factors activate transcription of target genes in concert and in association with coregulatory proteins, a class of proteins that do not bind to DNA themselves. The route from target gene binding by a transcription factor to gene transcription is a multistep process, which involves both recruitment and release of coregulators, chromatin remodeling and activation of the basal transcription machinery. Some coregulators, such as Tat-interactive protein-60KDa (Tip60), can alter the local chromatin context. Tip60 is a member of the MYST family of histone acetyltransferases, named after its founding members MOZ, Ybf2/Sas3, Sas2 and Tip60, which share a highly conserved MYST acetyltransferase domain, but display limited homology outside this region [5], [6]. Tip60 is the catalytic subunit of the highly conserved NuA4 acetyltransferase complex [7], [8], which plays a key role in transcription regulation, cell cycle and checkpoint control, apoptosis and DNA damage repair [9], [10], [11]. Tip60 can be recruited to the promoter of certain genes through transient interaction with a variety of different transcription factors, where it can acetylate histone proteins (H4, H2A, H2A.X and H2A.Z) and various transcription factors, thereby activating or repressing transcription [10]. Activation requires the HAT activity of Tip60, while repression is thought to be independent from its HAT activity and may result from its interaction with transcriptional silencers and histone deacetylases [10]. Tip60 transiently associates with a growing list of specific transcription factors where it acts either as a coactivator or as a corepressor. Recently, we identified Tip60 as a positive regulator of PPARγ transcriptional activity [12]. Tip60 interacts with the N-terminal AF1 region of PPARγ, a region of the protein implicated in isotype-selective gene expression and adipogenesis [13], [14]. Chromatin immunoprecipitation experiments showed that the endogenous Tip60 protein is recruited to PPARγ target genes in mature 3T3-L1 adipocytes, but not in pre-adipocytes, indicating that Tip60 requires PPARγ for its recruitment to PPARγ target genes [12]. Interestingly, expression of the Tip60 protein, but not mRNA, increases during the first stages of 3T3-L1 differentiation [12], suggesting that regulation of Tip60 protein levels may play an important role in early adipogenesis. Indeed, transcriptome analysis revealed several cell cycle genes to be regulated by Tip60, and knock down of Tip60 resulted in impaired mitotic clonal expansion, an early step in adipogenesis [15]. Together, these findings qualify the MYST acetyltransferase Tip60 as an adipogenic factor, that operates through two different mechanisms: in early adipogenesis it regulates several cell cycle genes during mitotic clonal expansion (MCE), while it functions as a PPARγ coactivator during the later stages of adipocyte differentiation. A role for Tip60 in adipocyte differentiation and/or function *in vivo* has however not been established so far. Here, we investigated the metabolic role of Tip60 *in vivo*, making use of heterozygous Tip60 knock out mice. Our data indicate that while heterozygous loss of Tip60 affects some metabolic parameters (caloric intake) and metabolic organs (liver weight), Tip60+/− mice display largely unaltered glucose metabolism.

## Materials and Methods

### Materials

The following antibodies were used: anti-Tip60 (sc-5725), Santa Cruz Biotechnologies; anti-tubulin (ab6046), Abcam; anti-rabbit-HRP (111035144) and anti-mouse-HRP (115035146), Jackson Immunoresearch Laboratories Inc. A custom-made Tip60 antibody has been described earlier [16].

### Animal studies

WT C57BL/6J mice (8 weeks; Charles River Laboratories) and Tip60^+/−^ mice [11], [17] that had been backcrossed to C57BL/6J for 12 generations, were age-matched and fed standard chow until age 11 weeks, and subsequently fed LFD (10% kcal% fat, Research Diet D12450B) or HFD (45% kcal% fat, Research Diet D12451) for 19 weeks. Intraperitoneal glucose tolerance test (IP-GTT) was performed as described [18]. In short, mice (age 29 weeks) were fasted overnight, glucose was injected intraperitoneally (0.5 g/kg body weight) and blood glucose levels were measured before, and at multiple time points after glucose injection (Accu-chek, Roche). All mouse study protocols were approved by the Utrecht University Ethical Committee for Animal Experimentation (protocol 2010.III.01.008) and were in accordance with current Dutch laws on animal experimentation.

### AT and liver immunohistochemistry and liver triglycerides

Hematoxylin and eosin (H&E) staining of adipose tissue and liver sections was performed using standard protocols. Liver triglycerides were determined in liver homogenates prepared in buffer containing 250 mM sucrose, 1 mM EDTA, and 10 mM Tris-HCl at pH 7.5 using a commercially available kit (Instruchemie, Delfzijl, The Netherlands) according to the manufacturer's instructions.

### RNA extraction, quantitative PCR and Western blot analysis

Snap-frozen epididymal adipose tissue was homogenized and RNA was extracted using Trizol (Invitrogen). RNA was purified on an RNeasy micro column (Qiagen), RNA integrity was checked with a Bioanalyzer (Agilent), and cDNA synthesis was performed with iScript (Bio-Rad). Quantitative PCR with SYBR Green (Bio-Rad) was run on a MyIq machine (Bio-Rad). Primers for quantitative RT-PCR were designed with the universal probe library (Roche); sequences were as follows: mTip60 Forw 5′-GCTGCTTATTGAGTTCAGCTATG-3′; mTip60 Rev 5′-GGATCTCCAAGATGGTTTGG-3′; m36B4 Forw 5′-AGCGCGTCCTGGCATTGTGTGG-3′; m36B4 Rev 5′-GGGCAGCAGTGGTGGCAGCAGC-3′.

For protein analysis, epididymal white adipose tissue (eWAT) was lysed with M-PER Mammalian Protein Extraction Reagent (Thermo Fisher Scientific, Etten-Leur, The Netherlands) and proteins were analyzed by Western blotting as described [12], [19]. Controls were generated by transient transfection of HEK293T cells (ATCC, Manassas, VA) with empty expression vector (pCDNA3.1) or an expression vector encoding HA-tagged Tip60 (isoform 2), as described [15].

## Results

### Tip60+/− mice have normal body weight despite higher food intake

As homozygous Tip60 null mice (Tip60−/−) are embryonic lethal around the blastocyst stage [11], [17], we used heterozygous Tip60 KO mice to investigate the metabolic role of Tip60. Tip60+/− mice, originally generated by replacing exons 1–9 with a neomycin-targeting vector and maintained on a 129sv-C57BL/6 mixed genetic background [11], [17], were first backcrossed to a C57BL/6 genetic background.

**Figure 1:**
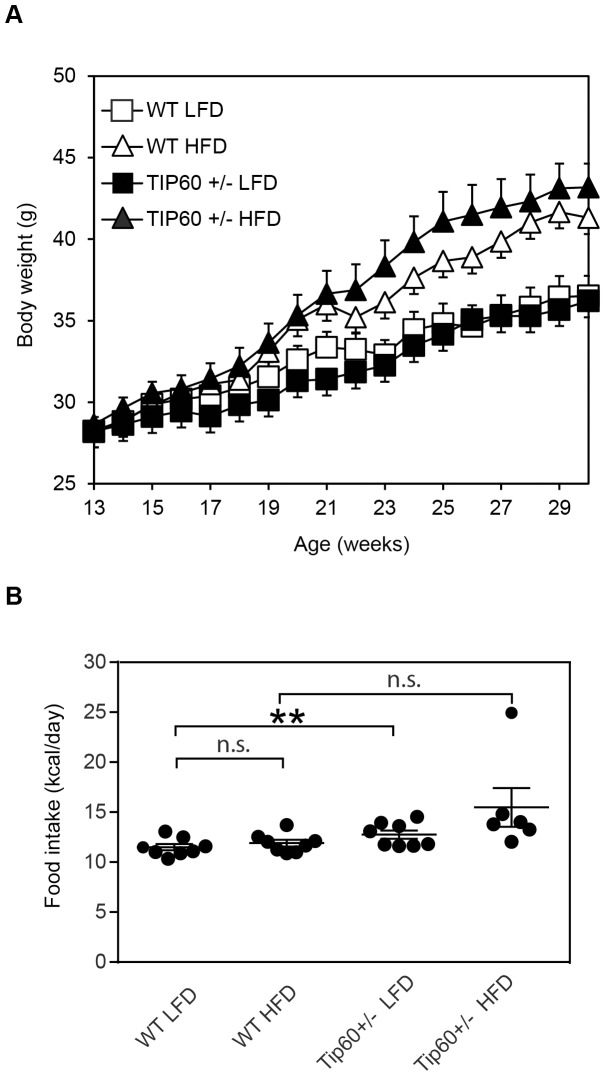
Tip60+/− mice display normal bodyweight with higher daily caloric intake. A, WT and Tip60+/− mice were weighed each week during 19 weeks of LFD or HFD feeding. Error bars represent means ± s.e.m. All groups contained 8 animals, except the Tip60+/− HFD group (n = 6). Please note slight reduction in weight around week 22 due to IP-GTT. B, Daily food intake of WT and Tip60+/− mice on LFD and HFD regimens. ** p<0.01, n.s. non-significant.

WT and Tip60+/− mice were fed a low fat diet (10% kcal% fat; LFD) or high fat diet (45% kcal% fat; HFD) for 19 weeks. While WT and Tip60+/− mice on HFD displayed significantly higher body weights compared to the LFD groups, no significant difference was observed between the genotypes (Fig. 1A). When caloric intake was analyzed, small differences were observed between the 4 groups (Fig. 1B). Taken together, these findings indicate that heterozygous Tip60 deletion may affect appetite (daily caloric intake) without affecting weight gain in a 19 weeks HFD regime.

### Heterozygous Tip60 deletion does not affect liver weight or insulin sensitivity

**Figure 2:**
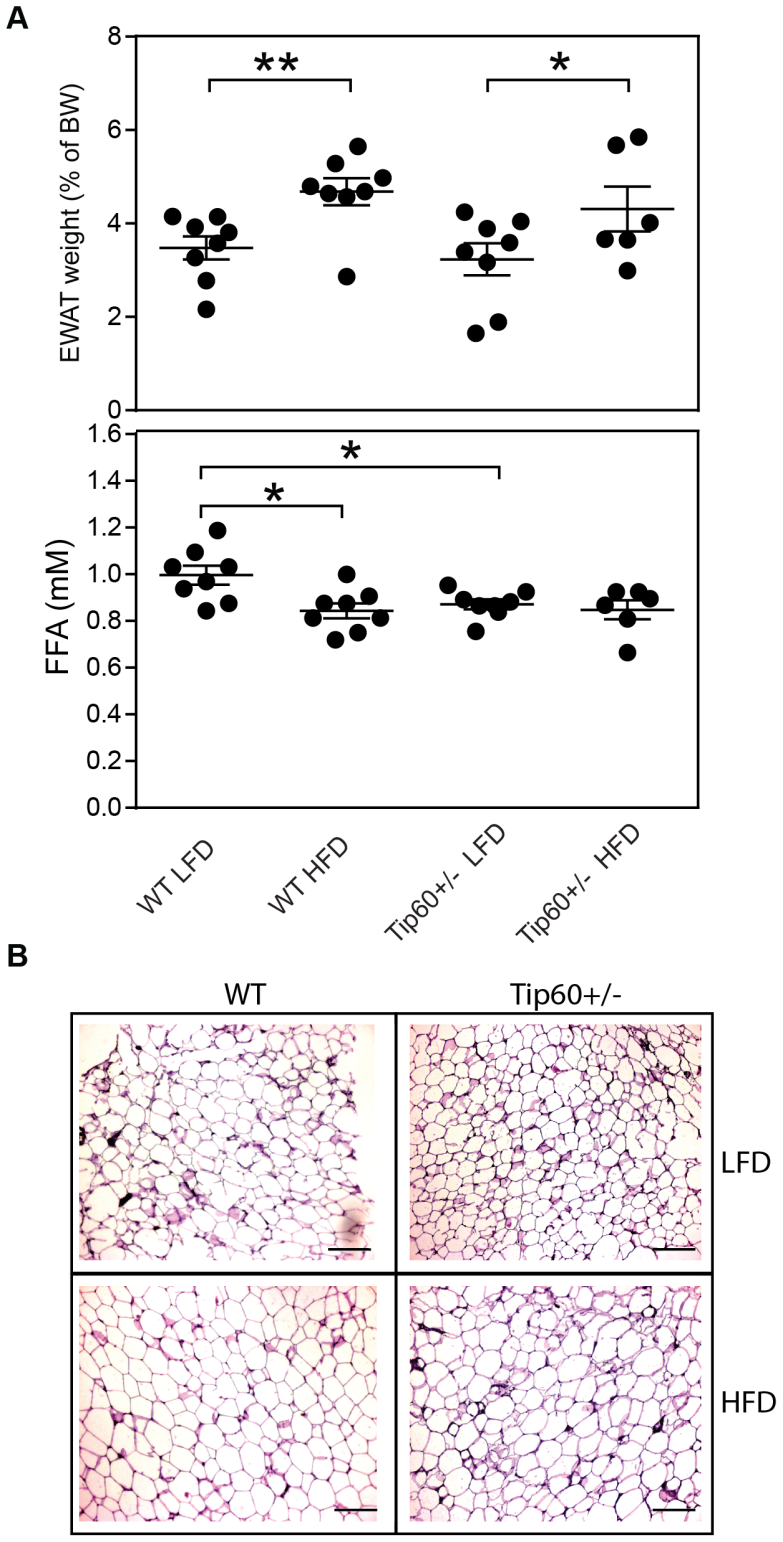
Tip60+/− mice display normal eWAT weight and morphology. A, Epididymal fat pad (eWAT) weights of WT and Tip60+/− mice as percentage of total body weight (BW) on a LFD and HFD regimen, measured after termination. * p<0.05, ** p<0.01. B, Plasma free fatty acid (FFA) levels. * p<0.05. C, H&E staining of representative eWAT sections from WT and Tip60+/− mice after 19 weeks of LFD or HFD feeding. Scale bars indicate 100 µm.

As knock down of Tip60 results in impaired adipogenesis in cultured cells[12], [20], we analyzed the epidydimal white adipose tissue (eWAT) depot of Tip60+/− mice on LFD and HFD. As shown in Fig. 2A, WT and Tip60+/− mice on HFD displayed significantly larger eWAT depots compared to the LFD groups, but no significant difference was observed between the genotypes. Small differences in circulating FFA levels were observed between the different animal groups (Fig. 2B). AT morphology was also clearly affected by HFD, with larger adipocytes, but not by genotype (Fig. 2C).

**Figure 3:**
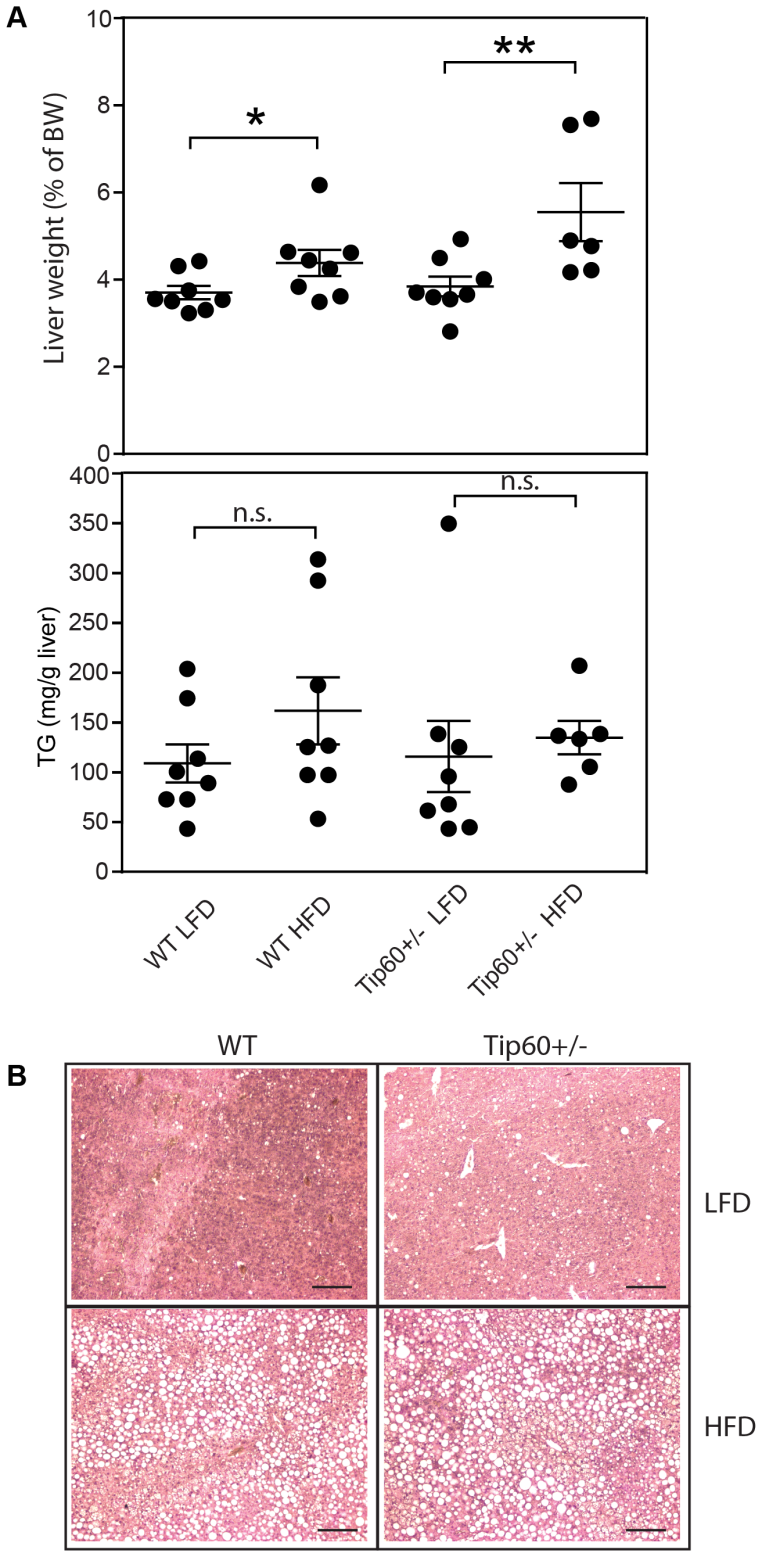
Tip60+/− mice display normal liver weight, TG content and morphology. A, Liver weight as percentage of total body weight (BW) of WT and Tip60+/− mice on a LFD and HFD regimen, measured after termination. * p<0.05, ** p<0.01. B, Liver triglyceride (TG) content. n.s. non-significant. C, H&E staining of representative liver sections of the WT and Tip60+/− mice fed LFD or HFD for 19 weeks. Scale bars indicate 100 µm.

When the liver weights were analyzed, significant differences were observed between the LFD and HFD groups, but not between WT and Tip60+/− animals (Fig. 3A). Liver triglyceride (TG) content was not significantly altered by diet or genotype (Fig. 3B). HFD feeding resulted in the formation of lipid droplets in the liver, but no additional effects were observed due to heterozygous Tip60 inactivation (Fig. 3C).

**Figure 4:**
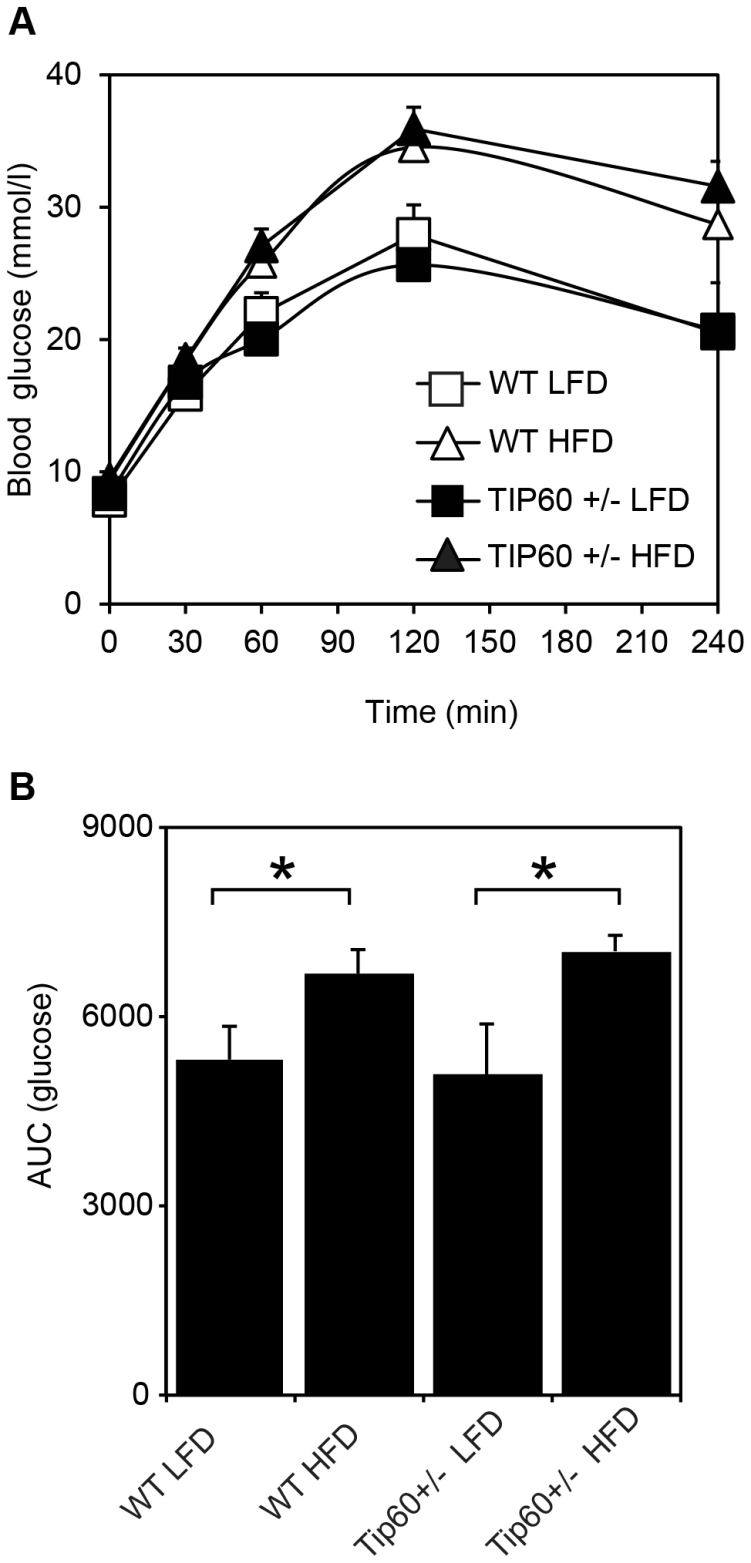
Tip60+/− mice display normal glucose tolerance. A,B, Intra-peritoneal glucose tolerance test was performed in WT and Tip60+/− mice after 18 weeks of LFD or HFD. All groups contained 8 animals, except the Tip60+/− HFD group (n = 6). Plasma glucose concentrations (A) and the area under the curve (B) for the various groups are shown. * p<0.05.

Next we analyzed whether glucose metabolism was affected by heterozygous deletion of Tip60. Glucose tolerance measured via an intraperitoneal glucose tolerance test (IP-GTT) was clearly impaired by HFD in WT and Tip60+/− mice, but no difference between the genotypes was observed (Fig. 4A, B). In summary, these results show that heterozygous inactivation of Tip60 has no dramatic effects on adipose tissue or liver, nor does it alter glucose tolerance.

### Reduced Tip60 mRNA but not protein expression in eWAT of Tip60^+/−^ mice

**Figure 5:**
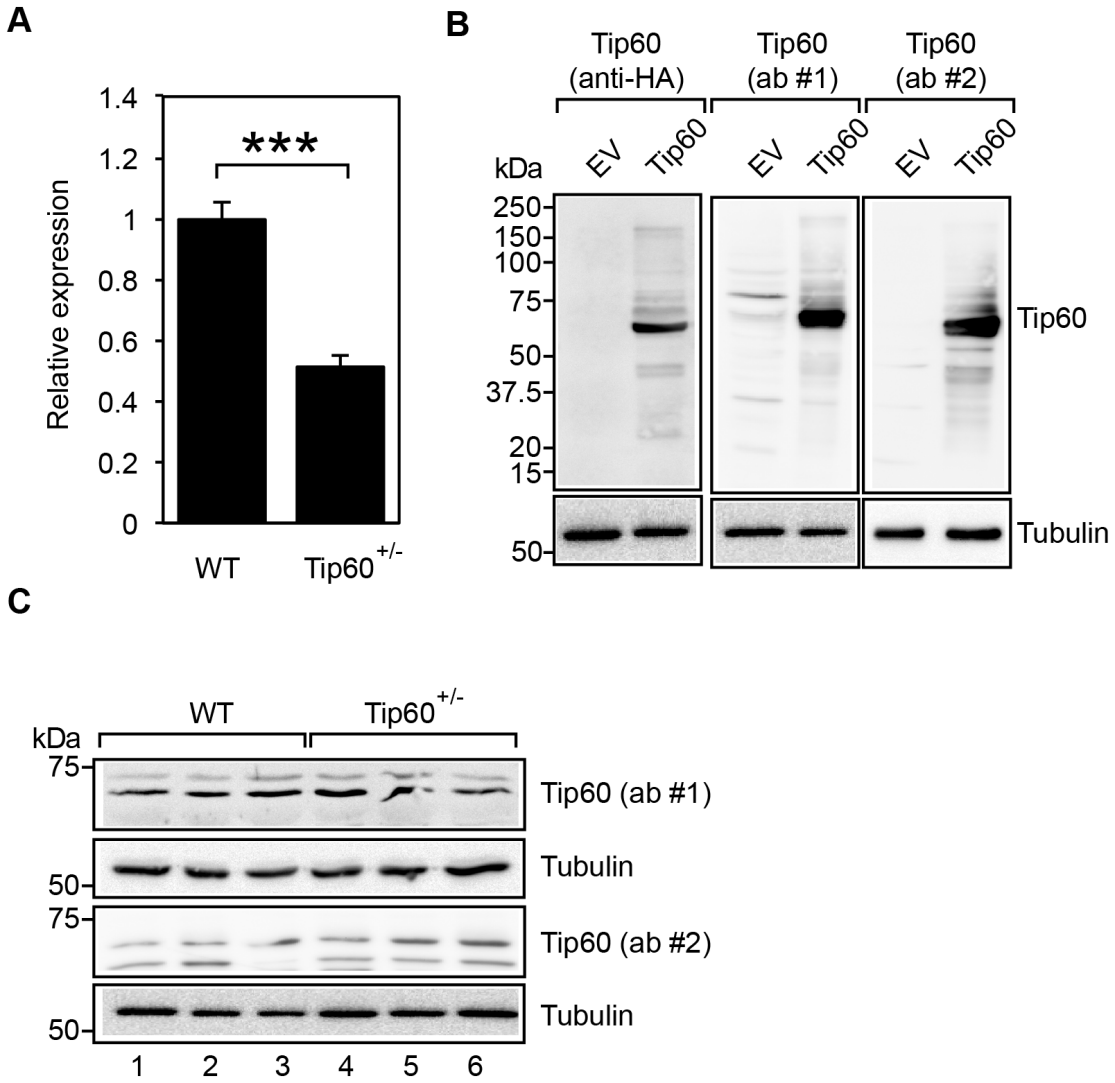
Tip60 mRNA, but not protein, is reduced in eWAT of Tip60+/− mice. A, Tip60 mRNA expression in eWAT of WT and Tip60+/− mice as determined by quantitative RT-PCR (n = 5 per group). Mean expression in WT mice was set at 1. *** p<0.001. B, HEK293T cells were transfected with HA-tagged Tip60 expression construct or empty vector (EV). Cell lysates were subjected to western blot analysis using an anti-HA antibody, a commercial Tip60 antibody (ab #1; sc-5725) or a custom-made Tip60 antibody (ab #2). Tubulin was used as a loading control. C, Tip60 protein expression in eWAT of WT (three individual animals; lanes 1–3) and Tip60+/− mice (lanes 4–6) as determined by Western blotting using 2 different antibodies against Tip60 (ab #1 and ab #2, as in panel B). Tubulin was used as a loading control.

Although knock down of Tip60 results in impaired adipogenesis in cultured murine 3T3-L1 preadipocytes [12] and human primary preadipocytes [20], no effect was observed due to heterozygous Tip60 deletion on total body weight (Fig. 1A) or eWAT depot weight (Fig. 2A). We therefore examined Tip60 mRNA and protein levels in WT and Tip60+/− mice. As expected, a 50% reduction of Tip60 mRNA levels was observed in eWAT of Tip60+/− mice compared to WT controls (Fig. 5A). Next, Tip60 protein expression in eWAT was analyzed by Western blotting, using a commercial antibody and a well-characterized custom-made Tip60 antibody [16]. Both antibodies specifically recognize overexpressed Tip60 protein (Fig. 5B), but the commercial antibody consistently detects higher molecular weight species of Tip60 (Fig. 5B, C), suggesting preferential recognition of proteins that underwent posttranslational modification(s) and/or specific Tip60 isoforms. Nonetheless, no significant differences in Tip60 protein levels were observed between WT and Tip60+/− mice with either antibody (Fig. 5C). These findings suggest that at least in eWAT the intact Tip60 allele probably compensates for the heterozygous loss of Tip60.

Taken together, our data indicate that while heterozygous inactivation of Tip60 affects food intake (Fig. 1B), Tip60+/− mice display largely unaltered overall glucose metabolism (Fig. 4). Heterozygous inactivation of Tip60 did not affect eWAT weight or histology (Fig. 2), which may be explained by compensation by the intact allele (Fig. 5).

## Discussion

Based on *in vitro* studies in murine 3T3-L1 preadipocytes [12] and human primary preadipocytes [20], the transcriptional coactivator and acetyltransferase Tip60 was identified as an essential adipogenic factor. We therefore investigated the role of Tip60 on adipocyte differentiation and function, and possible consequences on energy homeostasis, *in vivo*. As homozygous deletion of Tip60 results in embryonic lethality [11], [17], we made use of heterozygous knock out animals. We observed no significant differences in AT weight and morphology between Tip60+/− animals and WT controls, either under normal feeding conditions (LFD) or upon 19 weeks HFD challenge. Glucose tolerance (IP-GTT) and lipid metabolism (plasma FFA, liver TG content) were also not different between the groups, indicating that heterozygous deletion of Tip60 has no major effects on AT development and function. Unfortunately, while Tip60 mRNA expression levels were reduced by approximately 50% in eWAT of Tip60+/− animals, Tip60 protein expression levels were not significantly different between the two genotypes (Fig. 5). This phenomenon has previously been reported in other tissues including B cells [11], cerebellum [21], as well as heart, liver, brain, skeletal muscle and kidney [16]. Taken together these findings suggest that the intact Tip60 allele in heterozygous animals compensates for the ablated Tip60 allele in order to maintain normal cellular function. Although the rationale and mechanism for Tip60 allele compensation is of interest, the function of Tip60 protein in these various tissues unfortunately remains unanswered. Accordingly, our observations preclude any conclusion on the role of Tip60 in AT *in vivo* at present. Next to Tip60, several other transcriptional cofactors have been implicated in energy homeostasis, some of which play a role in adipocyte differentiation and/or function, including the coactivators SRC12, -2 and -3, CBP and p300, and the corepressors RIP140, NCoR and SMRT [22], [23]. The role of some of these factors in adipocyte differentiation and/or function has been studied *in vivo* (e.g. SRC1 and -2; [24], RIP140 [25]), but, like Tip60, whole body gene knock outs of several cofactor genes results in embryonic lethality (e.g. NCoR[26]; SMRT[27]; CBP and p300 [28]). Alternative approaches have therefore been used, including tissue-specific homozygous gene inactivation (e.g. [29]), or generation of animals bearing specific knockin mutations [30], [31], [32], [33]. Similar approaches are required to establish the role of Tip60 in AT development and function, and its subsequent role in energy homeostasis, *in vivo*. For this purpose, a line of mice bearing Lox-flanked Tip60 alleles is now available for tissue-specific targeting (J. Lough, personal communication).

Recently, Tip60 was implicated in gluconeogenesis [34]. Using yeast proteome microarrays, Lin *et al.* found the NuA4 complex to acetylate the yeast phosphoenolpyruvate carboxykinase enzyme (Pck1p), a rate-limiting enzyme in gluconeogenesis. Acetylation of Pck1p was crucial for enzymatic activity and the ability to grow on non-fermentable carbon sources [34]. In addition, Tip60-mediated acetylation of the mammalian homologue PEPCK may also be important for gluconeogenesis, as knock down of Tip60 in the human hepatoma cell line HepG2 resulted in reduced glucose secretion [34]. In our *in vivo* studies fasting glucose levels were unaltered in Tip60+/− mice (data not shown). It should be noted however that we did not specifically address hepatic gluconeogenesis. Furthermore, Tip60 protein expression levels may also be only marginally affected in the liver, analogous to our observations in eWAT (Fig. 5). Therefore, additional studies are required to address the potential role of Tip60 in gluconeogenesis *in vivo*.

Despite the fact that steady-state levels of Tip60 protein were unaltered in eWAT of Tip60+/− mice (Fig. 5), these animals display accelerated onset and enhanced penetrance of Myc-induced B-cell lymphomas when crossed with E_μ_-*myc* transgenic animals [11]. This phenotype as well as recently published findings that stress induces partial cell-cycle activation in Tip60 haploinsufficient adult cardiomyocytes [16] suggests that the intact Tip60 allele does not completely compensate for heterozygous inactivation of Tip60 when the animal is placed under biochemical stress mediated by transgenic over-expression of molecules such as Myc [11], or physical stress induced by aortic banding [16]. Also in our study Tip60+/− animals displayed a phenotypic alteration: heterozygous inactivation of Tip60 resulted in slightly higher daily caloric intake. The higher caloric intake did not result in increased body weight, suggesting that energy expenditure was increased. Future studies are required to establish whether this may be explained by reduced Tip60 protein levels in metabolic organs other than eWAT (i.e. brain, BAT) in Tip60+/− animals.
